# Immunotherapy as a neoadjuvant preoperative treatment for locally advanced pulmonary sarcomatoid carcinoma: a case report of clinical efficacy

**DOI:** 10.3389/fonc.2025.1483766

**Published:** 2025-01-29

**Authors:** Hanhan Li, Ruilian Chen, Wei Guo, Shang Xiang, Jiayang Huang, Hanrui Chen

**Affiliations:** ^1^ The First Affiliated Hospital of Guangzhou University of Chinese Medicine, Guangzhou University of Chinese Medicine, Guangzhou, Guangdong, China; ^2^ Lingnan Medical Research Center, Guangzhou University of Chinese Medicine, Guangzhou University of Chinese Medicine, Guangzhou, Guangdong, China

**Keywords:** pulmonary sarcomatoid carcinoma, rare lung cancer subtype, tumor immunotherapy, neoadjuvant treatment, pathological complete remission, case report

## Abstract

**Background:**

Pulmonary sarcomatoid carcinoma (PSC) is a rare subtype of non-small cell lung cancer (NSCLC) with a generally poor prognosis. Studies have shown that the survival rate for advanced-stage patients is less than 5%, with a median overall survival (OS) of less than 6.5 months.

**Case report:**

Herein, we report a case of locally advanced PSC in a 56-year-old male patient who received platinum-based double-drug therapy combined with anti-angiogenic drugs, followed by immunotherapy as the first-line systemic treatment. After undergoing eight cycles of neoadjuvant therapy, the opportunity for surgery arose. Postoperative pathology confirmed that the neoadjuvant chemotherapy in this regimen achieved pathological complete remission (pCR). At present, the patient maintains a good quality of life, continues with immunotherapy for consolidation and prevention of recurrence, and has reached a survival period of 10 months.

**Conclusion:**

For patients with locally advanced PSC who do not have significant genetic mutations, the use of immunotherapy as a first-line neoadjuvant and adjuvant therapy may hold promise for achieving a pCR. Further research into this treatment protocol is warranted.

## 1 Introduction

Pulmonary sarcomatoid carcinoma (PSC) is a rare subtype of non-small cell lung cancer (NSCLC) characterized by the presence of sarcomatoid components, such as spindles or giant cells, with a generally poor prognosis (1–3). It accounts for approximately 0.1%–0.4% of all NSCLC cases (4, 5). Due to its rarity, clinical trials for PSC have remained at the phase II stage (6), and specific diagnostic and therapeutic strategies have yet to be established. Based on recently reported cases, the treatment principles for PSC are similar to NSCLC, with surgery as the primary approach for early-stage patients (7, 8). However, due to the insidious onset and high invasiveness of PSC, most patients are diagnosed at an advanced stage, and PSC shows limited sensitivity to radiation and chemotherapy compared to other NSCLC subtypes, resulting in a poorer prognosis (9). Therefore, comprehensive treatments, including chemotherapy, radiotherapy, immunotherapy, and targeted therapy, are the mainstay for advanced-stage PSC patients (10–13). Studies have shown that the survival rate for advanced-stage patients is less than 5%, with a median overall survival (OS) of less than 6.5 months (14, 15).

Herein, we report a case of locally advanced PSC in a patient who received platinum-based double-drug therapy combined with anti-angiogenic drugs, followed by immunotherapy as the first-line systemic treatment. After undergoing eight cycles of neoadjuvant therapy, the opportunity for surgery arose. Postoperative pathology confirmed that the neoadjuvant chemotherapy in this regimen achieved pathological complete remission (pCR). At present, the patient maintains a good quality of life, continues with immunotherapy for consolidation and prevention of recurrence, and has reached a survival period of 10 months.

## 2 Case presentation

### 2.1 Chief complaints

A 56-year-old male patient was transferred to the oncology department with complaints of “pain in the right rib area and the discovery of a large mass in the upper right lung.”

### 2.2 History of present illness

**Figure 1:**
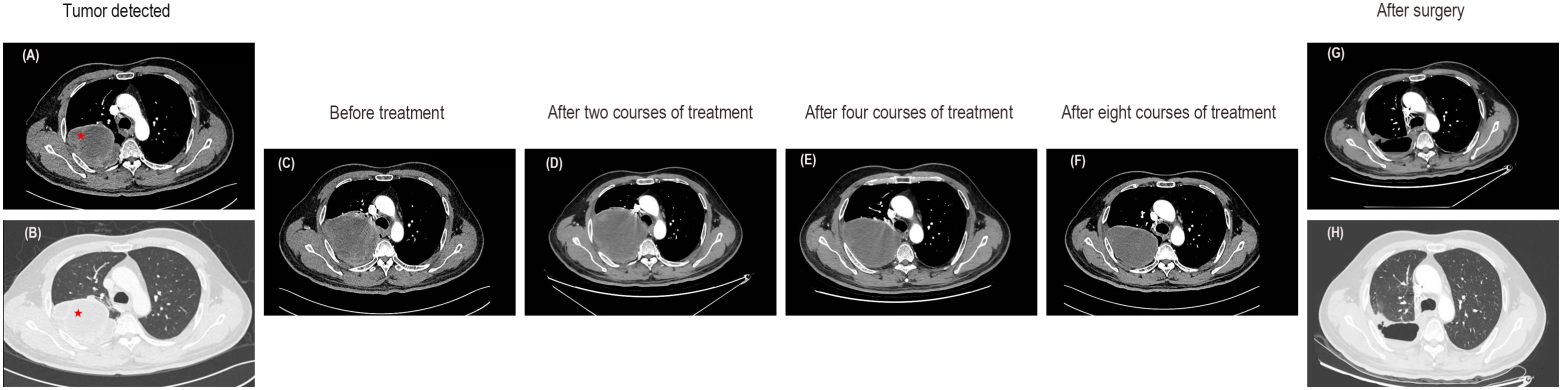
Enhanced CT images of the lung mass. **(A, B)** CT images at the time of tumor detection. **(C)** The CT images before any treatment. **(D)** Enhanced CT scans after two courses of treatment indicated a slightly enlarged tumor with an overall stable efficacy. **(E)** A subsequent CT scan after the fourth course of treatment showed stable disease. **(F)** The CT images after eight courses of treatment suggested a decrease in tumor size. **(G, H)** Enhanced CT revealed no tumor recurrence after surgery.

In November 2022, a chest computed tomography (CT) scan revealed a massive tumor in the right upper lobe measuring 91x80x137mm, raising suspicions of lung cancer. Enlargement of the right hilar and mediastinal lymph nodes was noted, along with a local invasion in the right 4th posterior rib (**Figures 1A, B**). The patient presented with normal mental status, right-sided rib pain, occasional bloody sputum in cough, and no complaints of wheezing or chest tightness. There had been no significant changes in recent body weight.

### 2.3 Physical examination

Diminished breath sounds in the upper right back of the lung and no dry or wet rales were heard in both lungs.

### 2.4 Pathological examination and clinical diagnosis

**Figure 2:**
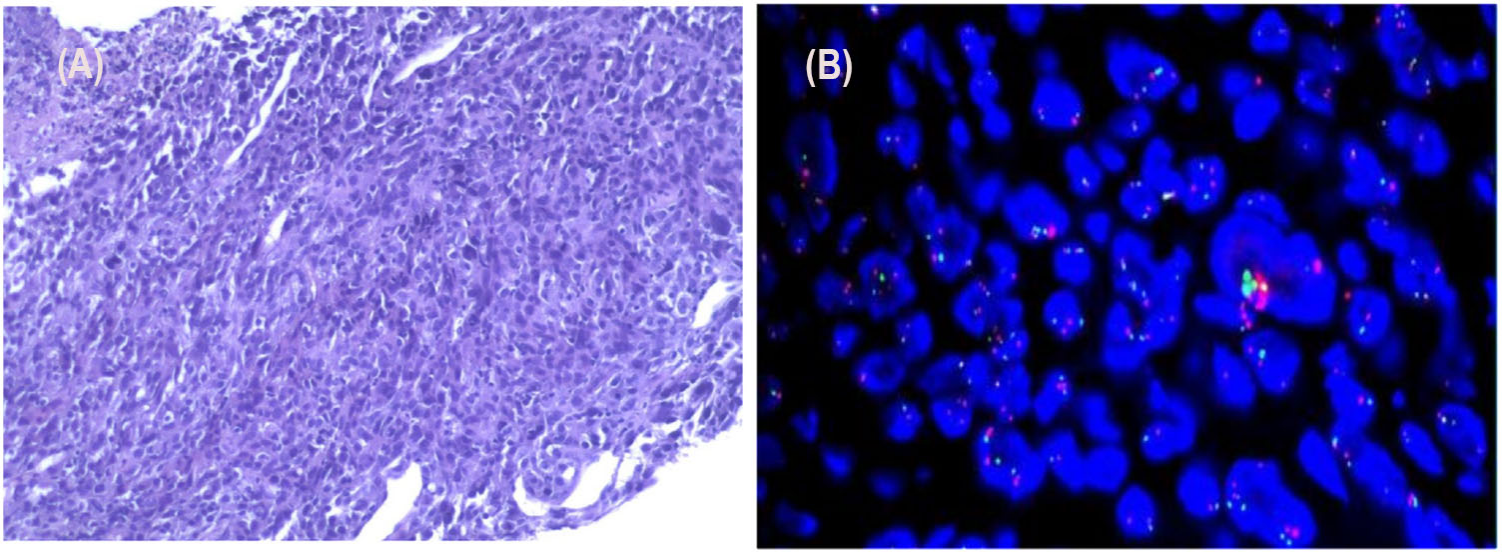
Pathology and fluorescence *in situ* hybridization (FISH) results of the lung aspiration biopsy. **(A)** Microscopic examination revealed spindle-shaped oncocytes arranged in bundles, oval-shaped nuclei, and frequent nuclear division. **(B)** FISH: FISH result (-), C-MET gene (no amplification), and C-MET signal exhibited a punctate distribution.

After the patient’s hospitalization, a lung mass puncture biopsy was performed. Microscopic examination revealed spindle-shaped oncocytes arranged in bundles, oval-shaped nuclei, and frequent nuclear division (**Figure 2A**). The immunohistochemistry results were as follows: CK (+), Vimentin (+), P40 (-), TTF-1 (-), Syn (-), SMARCA4 (+), ALK (D5F3, -), Ki-67 (70+), HER-2 (-; 10% of nuclei positive), PD-L1 (TPS: 40%). Fluorescence *in situ* hybridization (FISH): FISH result (-), C-MET gene (no amplification), and C-MET signal exhibited a punctate distribution (**Figure 2B**). The genetic testing analysis report indicates that the tumor mutation burden (TMB) was 21.83 Muts/Mb, with a TP53 mutation abundance of 60.48%; the KRAS mutation abundance was 30.66% and no other clinically significant driver gene mutations were detected. Based on laboratory tests, imaging examinations, and pathological findings, the diagnosis was a pulmonary malignant tumor (poorly differentiated sarcomatoid carcinoma, T4N1M0, stage IIIA).

### 2.5 Treatment

In this case, the patient presented with a massive right upper lung mass. An enhanced CT scan revealed an increase in the size of the right upper lobe pulmonary sarcomatoid carcinoma (107X95X160mm) compared to previous scans, along with enlarged right hilar and mediastinal lymph nodes, and slight progression, with local invasion into the chest wall and ribs, similar to previous findings (**Figure 1C**). Following consultation with thoracic surgeons, it was determined that, due to the tumor’s enormous size and local invasion into the chest wall and ribs, surgical intervention was not indicated at this time. Therefore, the initial treatment plan involved neoadjuvant chemotherapy, with the possibility of reassessing surgical options after tumor reduction. In terms of oncological treatment, a regimen combining chemotherapy and targeted therapy was considered, and further treatment options would be determined based on its effectiveness. With the patient’s and family’s consent, the first round of chemotherapy combined with targeted therapy was administered. The specific medications used were docetaxel (140mg on day 1) combined with cisplatin (50mg on days 1-2, 40mg on day 3) and bevacizumab(500mg on day 1), with every 21 days as one cycle. Starting in the second course of treatment and based on the genetic testing results and after consulting with the patient and family, the more cost-effective immune checkpoint inhibitor (ICI), tislelizumab (200mg on day 1), was ultimately selected. Thus, immunotherapy was introduced in addition to the existing chemotherapy and targeted therapy regimen. A follow-up CT scan showed stable disease (the tumor slightly increased in size to 114X104X160mm) (**Figure 1D**). During the fourth course of treatment, considering the side effects of docetaxel and cisplatin, the chemotherapy regimen was changed to a safer option, single-agent albumin-bound paclitaxel. The specific medications used were albumin-bound paclitaxel (400mg on day 1) combined with bevacizumab (500mg on day 1) and tislelizumab (200mg on day 1), with every 21 days as one cycle. A subsequent CT scan continued to show stable disease (a reduction in the size of the tumor to 109X90X152mm) (**Figure 1E**). During the fifth course of treatment, the original treatment plan was continued for one additional cycle. After completing six cycles of chemotherapy, the treatment was discontinued. Tislelizumab monotherapy was administered during the seventh and eighth courses of treatment. A follow-up CT scan showed stable disease (the size of the tumor was reduced to 100X76X128mm) and the majority of the mass appeared cystic without enhancement (**Figure 1F**). The CT scan demonstrated a reduction in tumor size with low-density areas and no enhancement within the tumor. Following a collaborative assessment by the radiologist and thoracic surgeon, there was a consensus that significant tissue necrosis was present. It was recommended that the patient undergo positron emission tomography-computed tomography (PET-CT) to assess the metabolic status of the tumor. However, the patient declined the examination due to financial reasons. Considering the patient’s current stable condition, it was determined that further conservative internal medicine treatment would not provide additional benefits. Therefore, after consultation with the thoracic surgery team, surgical treatment was recommended. With the patient’s consent, the patient underwent video-assisted thoracoscopic surgery.

### 2.6 Outcome and follow-up

**Figure 3:**
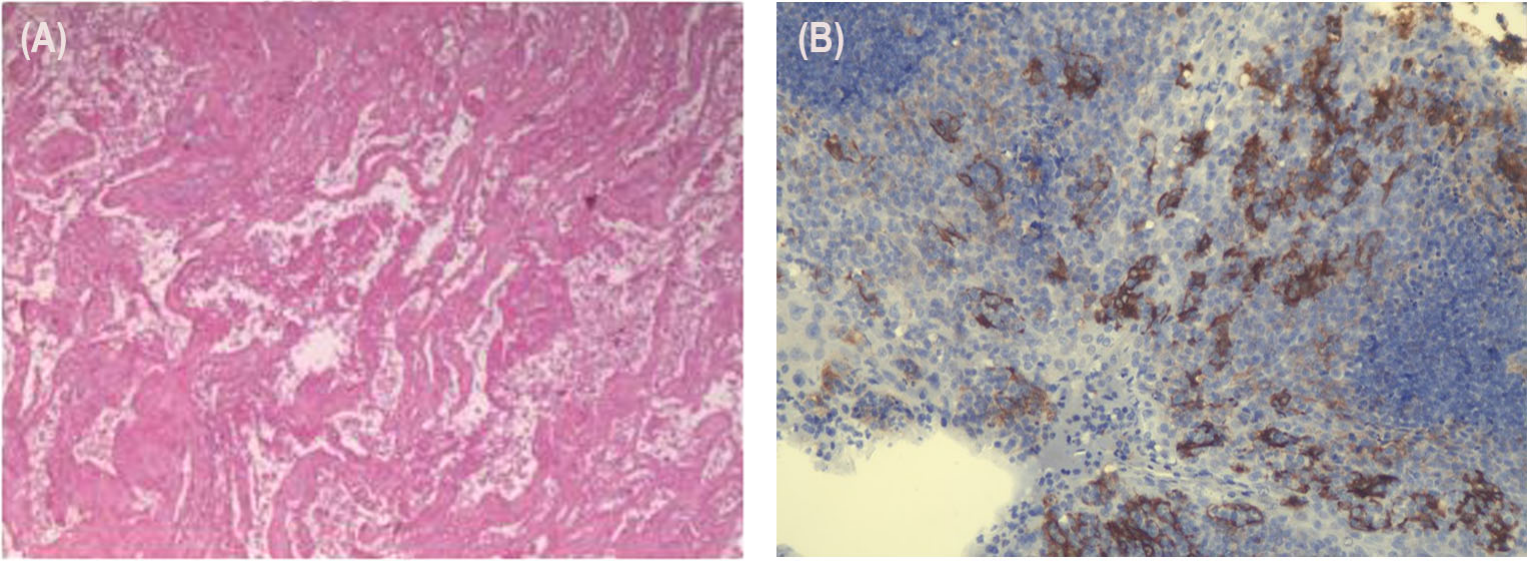
Tissue and pathology after resection of the lung cancer. **(A)** (1) In the right upper lung, consistent with changes after tumor chemoimmunotherapy, no residual viable tumor cells were found in the tumor bed. (2) There was diffuse congestion and hemorrhage in the peripheral lung tissue, with no tumor tissue observed at the bronchial margin. (3) No tumor metastasis was observed in the lymph nodes of groups 2, 4, 7, 10, and 11, and the subcarinal lymph node (0/3, 0/3, 0/1, 0/1, 0/2, 0/2). **(B)** In total, 40% of the tumor cells exhibited positive PD-L1 expression.

The postoperative pathology results were as follows: (1) In the right upper lung, consistent with changes after tumor chemoimmunotherapy, no residual viable tumor cells were found in the tumor bed. (2) There was diffuse congestion and hemorrhage in the peripheral lung tissue, with no tumor tissue observed at the bronchial margin. (3) No tumor metastasis was observed in the lymph nodes of groups 2, 4, 7, 10, and 11, and the subcarinal lymph node (0/3, 0/3, 0/1, 0/1, 0/2, 0/2) (**Figure 3A**). The immuno-histochemistry results of the surgical tissue indicate a PD-L1 (TPS of 40%) (**Figure 3B**).

**Figure 4:**
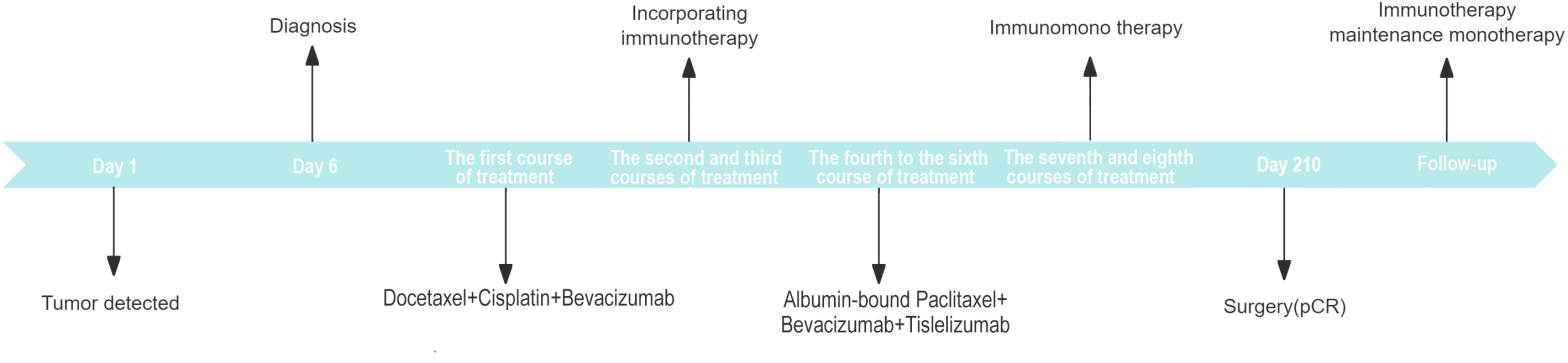
Diagnosis and treatment timeline.

Currently, the patient is in good physical condition and continues to attend regular outpatient follow-up visits. In the most recent follow-up at one month postoperatively, the CT scan showed the following findings: a right upper lobe pulmonary sarcomatoid carcinoma post-surgery, irregular band-like opacities in the surgical area, and thickening of the right interlobar fissure and pleura. Regular follow-up with CT or PET-CT scans is recommended. Rib changes were essentially unchanged from before and some mediastinal lymph nodes appeared slightly enlarged. Due to inflammation in the left lower lobe, a post-treatment follow-up was recommended (**Figures 1G, H**). To consolidate the treatment effect, immunotherapy was continued in the third month postoperatively with a regimen of tislelizumab. The patient’s overall diagnostic and treatment process is shown in **Figure 4**.

## 3 Discussion

Lung cancer is one of the most common malignancies worldwide. According to data from the 2020 Global Cancer Observatory project of the International Agency for Research on Cancer, there were approximately 2.2 million new cases of lung cancer and approximately 1.8 million deaths attributed to it. PSC represents a specific subtype within the category of NSCLC. PSC is recognized as a rare and highly aggressive malignancy when compared to other types of NSCLC. It demonstrates a higher degree of invasiveness and a tendency towards distant metastasis, and, coupled with the challenges in diagnosis, leads to a poorer prognosis relative to other NSCLCs. Data analysis from the National Cancer Database (NCDB) in the United States revealed that between 1998 and 2011, out of 1,547,531 NSCLC cases identified in the NCDB, 7,965 (0.5%) were confirmed as PSC. Among these, the survival rate for patients in advanced stages was found to be no more than 6 months (5). Lococo et al.’s research found that even among patients with PSC who underwent R0 resection at an early stage, there was still a recurrence rate of 62% (16). Moreover, there is a growing trend of younger individuals being affected by PSC, posing a significant threat to patients’ health and lives (3, 17).

The treatment principles for PSC are similar to those of NSCLC, where early-stage patients primarily undergo a comprehensive treatment approach with surgery as the mainstay. A retrospective analysis of 262 PSC patients at the China National Cancer Center found that patients who underwent surgical treatment had significantly improved median survival compared to those who did not (23.0 months vs. 11.0 months) (8). For advanced-stage patients who do not have the opportunity for surgery, the preferred treatment approach aligns with the first-line treatment for NSCLC patients without driver gene mutations. Some retrospective clinical studies have shown that advanced-stage patients can benefit from first-line chemotherapy (18). A study demonstrated that in 32 patients with pulmonary sarcomatoid carcinoma, the application of gemcitabine, cisplatin or paclitaxel, and cisplatin chemotherapy regimens achieved an objective response rate of 21.9%, with a median survival time of 14 months, showing efficacy similar to that observed in other non-small cell lung cancers (19). In a phase II prospective study reported in 2022, 16 patients with PSC were treated with carboplatin and paclitaxel (CP), with or without bevacizumab. The results indicated that the median progression-free survival for the CP group was 1.2 months, compared to 4.2 months for the CP and bevacizumab (CPB) group. Additionally, the median overall survival for the CP group was 7.9 months, while for the CPB group it was 11.2 months (20). However, overall, PSC shows a lower response to chemotherapy compared to other types of lung cancer. In this case, the patient responded well to chemotherapy, with a certain degree of tumor growth inhibition observed after the first two cycles of platinum-based regimens.

In the treatment of PSC, when meaningful gene mutations are present, targeted therapy takes precedence over immunotherapy. However, in this case, the patient did not exhibit mutations in the driver genes, making him ineligible for targeted therapy. Research by Vieira and colleagues has shown that PSC exhibits high PD-L1 overexpression, significant tumor microenvironment immune infiltration, and a high TMB compared to other NSCLC subtypes (21). This suggests that PSC may benefit from treatment with ICIs. A study from China included 37 PSC patients who received second-line or above ICI immunotherapy. Regardless of PD-L1 status, the overall remission rate was 40.5% and the disease control rate was 64.8%. The median overall survival was 12.7 months (ranging from 0.3 to 45.7 months) (22). A retrospective study involving 32 PSC patients found that 59.4% of them were TPS>1%. Furthermore, it demonstrated a positive correlation between PD-L1 expression and CD8+ T cell infiltration, suggesting that patients with both PD-L1+ and CD8+ T cells may benefit from single-agent ICI therapy (23). In a study by Qian and colleagues (12), immunotherapy, whether used as a monotherapy or in combination as a first-line treatment for PSC, effectively extended patients’ progression-free survival and overall survival without significant adverse reactions. Although current research on immunotherapy for PSC is predominantly retrospective or based on sporadic case reports (24, 25), some prospective clinical trials for PSC immunotherapy are underway (NCT02834013, NCT03022500, and NCT04224337, among others). Statistical studies have shown that adjuvant chemotherapy is an independent factor affecting the prognosis of surgical patients with PSC, with more significant benefits observed in advanced cancer cases, younger patients, or those with a higher body mass index (26). When analyzing the treatment process of this patient retrospectively, he was diagnosed at an advanced stage and was deemed to not be a candidate for surgery after assessment. The patient had positive PD-L1 expression and a TMB of 21.83 Muts/Mb. Multiple studies have indicated that patients with NSCLC who have either positive PD-L1 expression or TMB-H may benefit from immunotherapy (27, 28). After careful consideration, we decided to incorporate ICI immunotherapy at an early stage, and among the ICI drugs, we chose tislelizumab. Tislelizumab is a humanized IgG4 monoclonal antibody against PD-1 that is independently developed in China. Tislelizumab’s unique molecular binding mechanism and superior binding kinetics demonstrate more comprehensive blocking activity for the PD-1/PD-L1 pathway. Additionally, the Fc region of the antibody has undergone genetic engineering to eliminate antibody-dependent cellular phagocytosis, thus avoiding T cell exhaustion and the weakening of the efficacy of PD-1 antibodies that can be caused by this effect (29, 30). After the introduction of tislelizumab in the second course of treatment, the patient responded well, with a significant reduction in tumor size. During the later maintenance phase of the single-agent immunotherapy, the tumor continued to shrink. After surgical treatment, a pathological examination showed no residual tumor and there was no lymph node metastasis, achieving pCR. Post-surgery, the patient’s general condition recovered well with a Performance Status score (PS score) of 90, indicating that neoadjuvant immunotherapy may benefit locally advanced PSC patients who are eligible for surgery.

This case also has certain limitations. These include the fact that PET-CT was not performed on this patient before surgery, making it impossible to assess the overall condition of the tumor. This is a report of a good response to the treatment of PSC with chemotherapy combined with antiangiogenic drugs and tislelizumab, indicating that immunotherapy may be a potentially promising strategy for the treatment of PSC. However, its effectiveness and safety need to be further verified in more cases and a more accurately targeted patient population needs to be selected.

## 4 Conclusion

For patients with locally advanced PSC who do not have significant genetic mutations, the use of immunotherapy as a first-line neoadjuvant and adjuvant therapy may hold promise for achieving a pCR. Further research into this treatment protocol is warranted.

## Data Availability

The original contributions presented in the study are included in the article/supplementary material. Further inquiries can be directed to the corresponding author.
